# Encouraging a Generation of Tremor Researchers: Macdonald Critchley's Paper on Essential Tremor

**DOI:** 10.1002/mdc3.13333

**Published:** 2021-09-08

**Authors:** Fabian Buechele, Christian R. Baumann, Andrew Lees, Günther Deuschl

**Affiliations:** ^1^ Department of Neurology University Hospital Zürich, University of Zürich Zürich; ^2^ University College London; and Reta Lila Weston Institute of Neurological Studies London United Kingdom; ^3^ Department of Neurology Universitätsklinikum Schleswig‐Holstein, Kiel Campus, Christian‐Albrechts University Kiel Germany

Critchley M. 
Observations on essential (heredofamial) tremor
. Brain 1949;72(Pt. 2):113–139.10.1093/brain/72.2.11318136705

Our choice for the most influential and important contribution of the second half of the last century about essential tremor (ET) is Professor Macdonald Critchley's paper published 1949 in Brain on “Observations on essential (heredofamilial) tremor”.^1^ The simple reason is that it summarizes for the first time the clinical presentation of this very common condition and discusses the related contemporary knowledge. Thereby, he has laid the foundation for clinical tremor research starting in the 1970‐ies of the last century.

Macdonald Critchley (1900–1997) has been the prototype of a British Neurology Professor of the past century (Fig. 1). Andrew Lees, one of his pupils at Queen Square Hospital in London, described him as “ascetic yet charismatic, tall and always impeccably dressed”^2^ with black suit and tie but certainly without lab coat. A brilliant and precise thinking on the one hand and a distant and mildly arrogant social interaction on the other hand belonged to this stereotype which is nowadays only rarely found at the legendary Queen Square Hospital. As one of the Queen Square deans (1948–1953) he was internationally highly regarded and traveled much more than others. He was elected President of the World Federation of Neurology (1965–1973). But at home he “only” became “commander of the British Empire” while his peers were named “knights” (Sir Francis Walshe, Sir Charles Putnam Symonds). In the contemporary British thinking this was—and still is—meaningful. We owe this outstanding neurologist the—at this time remarkable—amount of 300 single authored papers which are mostly of high quality and often entered non‐mainstream territories. We can only speculate that writing his paper on the neurology of old age in 1931^3^ has evoked his interest in essential tremor.

**FIG. 1:**
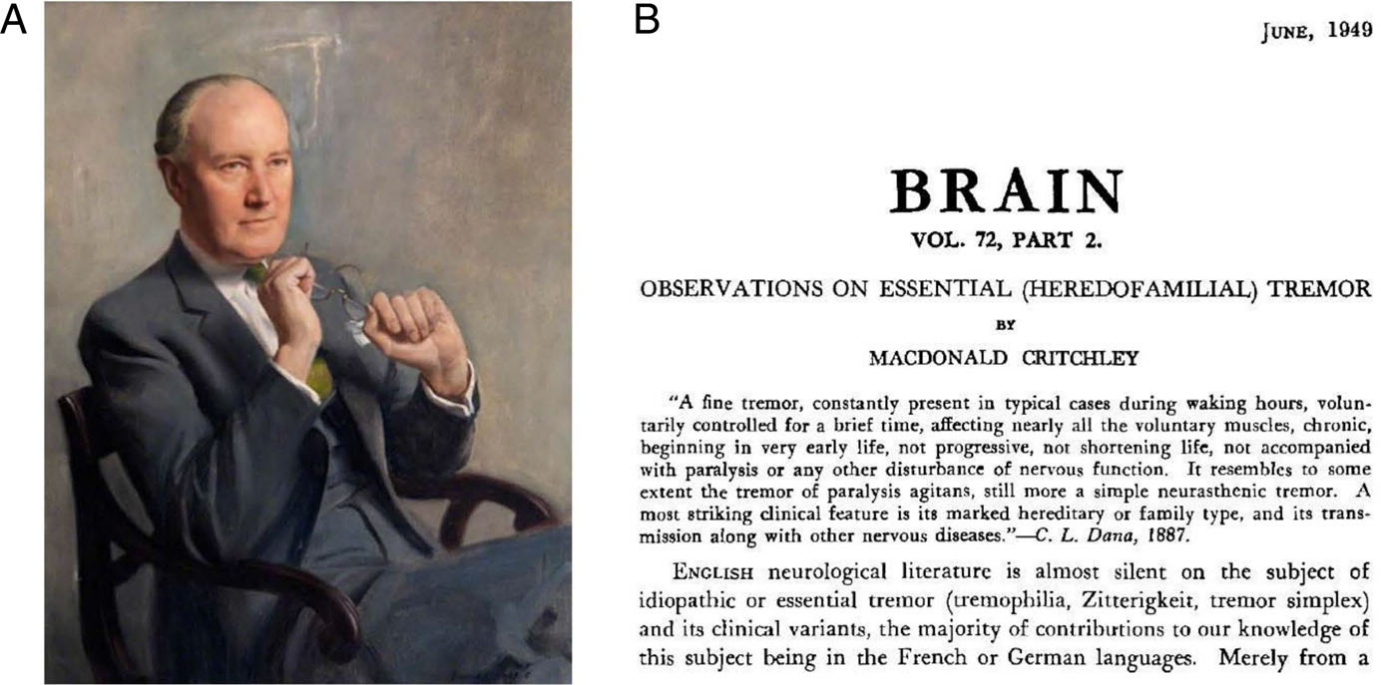
Portrait of professor Macdonald Critchley (**A**) showing him at the time when he was dean of the Queens Square hospital. The painting is nowadays hanging in the Wolfson lecture theater at the National Hospital, Queen Square, London). The first page of his brain paper published in 1949 (**B**).

The paper's main achievement is the creation of a positive definition of ET as a specific clinically relevant entity. In the first half of the 20th century, ET was mainly covered by case reports. The only two extensive publications addressing the issue more globally were the 264‐pages monography of Josef Pelnar in 1913 from Prague (Czech Republic) on “Das Zittern”^4^ and the 1941 “Habilitationsschrift” of Richard Jung^5^ from Freiburg (Germany) on “Physiological studies of Parkinsonian and other tremors” which addressed ET mainly to delineate it from other forms of tremor.

One of the highlights of Critchley's manuscript is the careful description of the clinical features of ET. Most importantly, he emphasized it as a monosymptomatic tremor disorder, mainly of the hands but possibly involving also other body parts, which remains the key feature in today's definition. Furthermore, he mentioned the wide range of age at onset (from childhood to senility), the almost equal distribution between male and female and the usually slow progressive clinical course although the symptom progression may come to a temporary standstill for many years. Furthermore, he described the topographic distribution affecting hands, head and voice in a descending frequency and points out that a unilateral manifestation is rare. The paper also recognizes that cranial involvement is a diagnostically helpful hint for ET compared to the rare occurrence of head tremor in Parkinson's disease. It nicely describes the different tremor frequencies occurring in ET and their limited value to separate the different tremors. It is also noted that “…some patients find that heavy dose of spirits will temporarily check tremor…” and that alcohol withdrawal can worsen tremor. The pharmacological effect of alcohol on GABA‐receptors as the pathophysiological background was unclear at that time.^6^ The publication describes the great influence of external factors such as fatigue or extreme temperatures on tremor severity as well as the ability of some patients to control their tremor during delicate movements, a feature which is not infrequently seen but definitely understudied until today.

Interestingly, Critchley separates essential tremor into mild high frequency tremor (resembling thyrotoxic or psychogenic tremor), medium frequency tremor (resembling Parkinsonian tremor) and even low‐frequency intention tremor. Charles D. Marsden later adapted this subdivision into his clinical classification of the variants of essential tremor^7^ which is nowadays no longer used.

With regard to additional signs and symptoms, Critchley did not yet separate essential from other forms of tremor including dystonic tremor but described many subtle accompanying symptoms in patients with ET and introduced them as “other involuntary movements” like choreiform contractions of the face or head or facial twitching. Nowadays, these particular soft signs are frequently recognized within the context of the special syndrome ETplus.^8^ Despite the frequent misunderstanding^9^ of this novel concept (ETplus) as a disease entity instead of a syndrome,^8^ these signs and symptoms have regained scientific interest^10^, ^11^ as they may indicate special variants with possibly different disease courses and various underlying etiologies. Interestingly, the notion that ET can develop into another disorder (eg Parkinson's) was already propagated by Critchley.

The paper does not explicitly mention non‐motor symptoms as part of the clinical presentation of ET, but extensively quotes other neurologists who reported that ET‐patients are often clinically presenting with a névrose trémulante (C. Achard) or psychical degeneration associated with a neuropathic stock (F. Raymond). Pelnar believed that ET‐patients were characterized by irritability, anxiety, shyness eccentricity, irresolution and other neurotic, hysterical and psychotic traits. Nowadays, we can only speculate that these figurative descriptions are summarizing the average personality profile of ET‐patients which has more recently been captured in recent studies as a tender‐minded^12^ and harm‐avoiding^13^ behavior. This symptom complex is nowadays mostly interpreted as a consequence of slight cerebellar abnormalities^14^ in ET and is currently discussed as the cerebellar cognitive affective syndrome.^12^

Regarding etiology, Critchley's paper nicely describes the obvious hereditary nature of ET with dominant inheritance pattern. Interestingly, the “phenomenon of anticipation” is proposed for ET meaning that the condition manifests earlier in life of every successive generation—a finding which has not been confirmed by modern genetics. With the rise of genetic sciences at the end of the last century there was a clear hope that the mystery of the origin of ET will be lifted in short time.^15^ Meanwhile, experts are struck by the lack of positive genetic findings despite intense research.^16^

Regarding pathology, inconsistencies are reported with some cases of basal ganglia or cerebellar abnormalities but normal findings in most other cases. Critchley therefore concluded “that it cannot be claimed that our knowledge of the pathology … is much advanced…”. It remains somewhat frustrating that this conclusion has not changed substantially until today: Current pathological studies are controversial as one group found a Purkinje‐cell loss in the cerebellum^17^ which could not be reproduced by two other groups.^18^, ^19^

Critchley's considerations on the “nature” of essential tremor are of particular interest. He initiates the discussion by putting ET into an almost philosophical perspective: “A life‐long monosymptomatic affection can scarcely be regarded as falling within the province of a morbid entity, any more than any other inborn and inherited peculiarity of physique or coloring” and elsewhere “In its nature as a constitutional monosymptomatic peculiarity it can scarcely be regarded as a “disease” and some medical men may never have been confronted with an example.’ This view is true in the sense that most patients with ET do not seek the help of physicians as observed in cross‐sectional studies.^20^, ^21^ But on the other hand, a significant proportion of these patients do have a severe handicap and benefit medical or even neurosurgical interventions.

At Critchley's time two pathogenetic interpretations of the condition were proposed. Lazar S. Minor (Vilnius/Moscow(Paris/Berlin, 1855–1942), proposed that ET is characterized by a “status macrobioticus multiparus,” a triad of tremor, longevity and fecundity, which he observed in his patients. Critchley was not convinced and discussed the controversial literature. Since then, there are two studies reporting that ET families^22^ and early‐onset ET patients^23^ have a longer life expectancy but larger series are lacking. On the contrary, several studies have shown on group level that at least elderly ET‐patients suffer from more cognitive deficits than controls. Those are usually mild^24^ and the profile of their dementia may be related to the cerebellar disturbance which was suggested first for motor symptoms of ET^25^, ^26^ and later for cognitive disturbances presumably corresponding to the “cognitive affective syndrome” of Schmahmann.^27^ Today, essential tremor is sometimes referred to as a “neurodegenerative disease” and is put on par with Alzheimer's or Parkinson's disease.^28^ Given the obviously different course of these conditions^29^ and the lack of consented data, Critchley would certainly have been skeptical of such interpretations.

On the contrary, Critchley discussed the concept of ET as “a degenerative disorder associated with a neuropathic family taint.” This is not corresponding to our present understanding of neurodegeneration but meant the occurrence of other neuropsychiatric signs and symptoms in the patients and their families like the occurrence of “nervousness,” “anxiety states,” “epilepsies” which were believed to be hereditary until the 1950‐ies. In his figurative language he considered ET as an “instance of an unmasking of an inherent property of neurocellular activity, as the result of some constitutional defect in the usual controlling mechanism”^30^ The pathogenesis was thereby interpreted as a loss of a control mechanism. Despite the common complaint of tremor he did clearly separate Parkinson's disease from ET.

This brief summary of Critchley's contribution shows how much was already known about this mysterious syndrome in 1950. His merit was a brilliant summary of the knowledge until then. We also can see how much has been added since then. PubMed teaches us that since 1950 more than 2500 papers have been published on ET and fundamental additions have been made in the past 70 years. When reading it carefully it reminds us of core questions for this syndrome which should guide our research.

## Author Roles

(1) Research project: A. Conception, B. Organization, C. Execution; (2) Statistical Analysis: A. Design, B. Execution, C. Review and Critique; (3) Manuscript Preparation: A. Writing of the first draft, B. Review and Critique.

F.B.: 1A, 1B, 3A

C.R.B.: 1A, 3B

A.L.: 1C, 3B

G.D.: 1A, 1B, 1C, 3B

## Disclosures

### Ethical Compliance Statement

This work did not require the approval of an institutional review board or informed patient consent. The author confirms that he has read the Journal's position on issues involved in ethical publication and affirms that this work is consistent with those guidelines.

### Funding Sources and Conflicts of Interest

No specific funding was received for this work. The authors have no conflicts of interest relevant to this work.

### Financial Disclosures for the Previous 12 Months

FB and CRB report no conflicts of interest. GD reports personal fees from Boston Scientific, Cavion, Aleva, Functional Neuromodulation and Thieme publishers.
